# Vegetable Grafting From a Molecular Point of View: The Involvement of Epigenetics in Rootstock-Scion Interactions

**DOI:** 10.3389/fpls.2020.621999

**Published:** 2021-01-07

**Authors:** Aphrodite Tsaballa, Aliki Xanthopoulou, Panagiotis Madesis, Athanasios Tsaftaris, Irini Nianiou-Obeidat

**Affiliations:** ^1^Institute of Plant Breeding and Genetic Resources, Hellenic Agricultural Organization (ELGO-Dimitra), Thessaloniki, Greece; ^2^Laboratory of Molecular Biology of Plants, School of Agricultural Sciences, University of Thessaly, Volos, Greece; ^3^Institute of Applied Biosciences, Centre for Research & Technology Hellas, Thessaloniki, Greece; ^4^Perrotis College, American Farm School, Thessaloniki, Greece; ^5^Laboratory of Genetics and Plant Breeding, School of Agriculture, Forestry and Natural Environment, Aristotle University of Thessaloniki, Thessaloniki, Greece

**Keywords:** epigenetics (DNA methylation), small RNAs (sRNAs), gene expression, vegetable grafting, rootstock-scion interaction

## Abstract

Vegetable grafting is extensively used today in agricultural production to control soil-borne pathogens, abiotic and biotic stresses and to improve phenotypic characteristics of the scion. Commercial vegetable grafting is currently practiced in tomato, watermelon, melon, eggplant, cucumber, and pepper. It is also regarded as a rapid alternative to the relatively slow approach of breeding for increased environmental-stress tolerance of fruit vegetables. However, even though grafting has been used for centuries, until today, there are still many issues that have not been elucidated. This review will emphasize on the important mechanisms taking place during grafting, especially the genomic interactions between grafting partners and the impact of rootstocks in scion’s performance. Special emphasis will be drawn on the relation between vegetable grafting, epigenetics, and the changes in morphology and quality of the products. Recent advances in plant science such as next-generation sequencing provide new information regarding the molecular interactions between rootstock and scion. It is now evidenced that genetic exchange is happening across grafting junctions between rootstock and scion, potentially affecting grafting-mediated effects already recorded in grafted plants. Furthermore, significant changes in DNA methylation are recorded in grafted scions, suggesting that these epigenetic mechanisms could be implicated in grafting effects. In this aspect, we also discuss the process and the molecular aspects of rootstock scion communication. Finally, we provide with an extensive overview of gene expression changes recorded in grafted plants and how these are related to the phenotypic changes observed. Τhis review finally seeks to elucidate the dynamics of rootstock-scion interactions and thus stimulate more research on grafting in the future. In a future where sustainable agricultural production is the way forward, grafting could play an important role to develop products of higher yield and quality in a safe and “green” way.

## Introduction

In a world where new pests are emerging every day and climate change alters the environment, food production in the future will be challenging. Plant grafting is one of the most valuable tools we have in vegetable production against soil-borne diseases and stresses. However, the benefits from using grafted plants are more; higher yields under stressful conditions, extension of the cultivation period, lower use of fertilizers and agrochemicals, broad use of phytogenetic resources as rootstocks, and no need for crop rotation are some of them (Lee et al., 2010).

Grafting was introduced from East Asia to Europe during the 20th century, but it has become more popular during the past 30 years. Grafted plants are commonly used today in the commercial production of tomato (*Solanum lycopersicum*), watermelon (*Citrullus lanatus*), melon (*Cucumis melo*), eggplant (*Solanum melongena*), cucumber (*Cucumis sativus*), pepper (*Capsicum annuum*), and many more. It is estimated that more than 10 million grafted tomato plants are used in greenhouses in United States while approximately 6 million are planted in open fields. Recognizing the emerging role of plant grafting in vegetable production, USDA established a grafting project for the improvement of science and use of vegetable grafting, involving academics from 10 United States universities.^1^

It is still unknown how plant grafting was discovered but it is likely that it originated from the occurrence of grafting in nature when two different plants come randomly in contact and unite their limbs or roots without human intervention. However, in this review, we only refer to grafting due to human intervention; it was employed to modify certain growth habits and characteristics or to bring disease resistance to pathogens. But why do we need to graft vegetables? The main reason is to fight soil-borne pathogens such as nematodes, fungi, bacteria, or viruses, especially after the ban of effective soil fumigants like methyl bromide and the restriction of pesticides. Although there are some alternatives, plant grafting appears as the most effective and sustainable solution to the problem. Use of resistant rootstocks that are intra-specific (within the same species) selections and have resistance genes or inter-specific (different species) and inter-generic (different genera) with non-host resistance mechanisms or have a resistance that lies on multiple genes, seem to be an important weapon in the fight against stresses (Louws et al., 2010). Furthermore, grafted plants are used against a variety of abiotic unfavorable soil conditions such as low water availability and drought (Rouphael et al., 2008; Sánchez-Rodríguez et al., 2012; Liu et al., 2016b; Zhang et al., 2020), flooding (Yetisir et al., 2006; Bhatt et al., 2015), nutrients deficiency (Zhang et al., 2012; Huang et al., 2016; Martínez-Andújar et al., 2017), high salt concentrations (He et al., 2009; Huang et al., 2011; Colla et al., 2012), heavy metals toxicity (Arao et al., 2008), and low or high temperatures (Rivero et al., 2003; Zhou et al., 2007; Venema et al., 2008). Finally, grafting has been used extensively in plant biology and physiology to identify mobile molecules, such as proteins, mRNAs, and small RNAs, that control important aspects of plant development and performance.

However, the molecular aspects of vegetable grafting remain vastly understudied. In this review, we are discussing the recent developments and findings on important mechanisms taking place during grafting, such as tissue reconnection and vasculature formation, rootstock-scion communication, and genomic interactions, and the impact of rootstocks in scion’s performance. Special emphasis will be drawn on the relation between vegetable grafting and epigenetics. Epigenetics involves stable and possible heritable gene expression alterations that are caused by how the DNA is packaged instead of changes of the primary DNA sequence (Bender, 2002). Epigenetics has been linked to almost every aspect of plant development and plant interactions with the environment. Currently, plant breeders can increase and use epigenomic variability through the use of genome-wide mapping of epigenetic marks and epigenetic target identification, in an effort to select new climate-smart crop varieties that are more resistant to environmental changes (Varotto et al., 2020). Whether plant grafting induces heritable epigenetic changes that have a significant impact on gene expression variability and subsequently on grafted plants’ phenotypes is an obvious question to be asked. Although this review will focus on vegetable grafting, a significant amount of related work has been done in model species like *Arabidopsis* or tobacco (*Nicotiana tabacum*), so these works will be discussed.

Certainly, the production cost of grafted plants is higher than the conventional. This is because manual grafting is highly labor-intensive as it demands highly skilled staff often working through a narrow time planting window to achieve high numbers of plants being grafted (Kubota et al., 2017). This problem can be addressed using automated grafting machines and robots or other cultivation practices that could significantly lower the prices of grafted plants. However, the amelioration of the grafting technique requires further research on the molecular mechanisms that govern grafted plants performance and significantly impact the phenotype. Work on the effects of grafting and rootstock-scion interactions is expected to shed more light into the complex interplay of grafting partners and the effect of this interplay into scion yield and quality.

## Grafting Technics and Long-Distance Signaling

Grafting involves joining cut tissues of two different plants to fuse into a single plant, sharing a unified vascular system. The re-establishment of the new plant entity starts with tissue connection between the rootstock and the scion at the grafting points, it proceeds with a vigorous cell division phase that results in the formation of a callus and common cell wall and it ends with the establishment of a unique vasculature system (Yin et al., 2012; Melnyk et al., 2015). In *Arabidopsis*, these grafting-related procedures are quite rapid, completed in only 1 week after grafting (Melnyk et al., 2015; Melnyk, 2017). The first that reconnects is the phloem, then root growth restarts, and in the end, xylem is reconnected (Melnyk et al., 2015). The attachment of the two plant parts together is vital as the root quickly responses transcriptionally to the presence of the shoot even if vascular connection has not been established yet (Melnyk et al., 2018). Plasmodesmata are formed between the adhering cells of the grafted plant establishing transport and cell-to-cell communication between the two grafting partners (Jeffree and Yeoman, 1983; Kollmann and Glockmann, 1985; Yin et al., 2012).

Grafting has been reported to be most successful when members of the same plant family are brought together to graft. An intriguing example is the Solanaceae family where successful grafts have been commercially developed between either tomato or eggplant with potato producing effectively edible fruits from both the rootstock and the scion (TomTato® and Egg and Chips® of Thompson and Morgan). However, it was recently proven that plants from different families even distant ones can be grafted successfully (Notaguchi et al., 2020); phylogenetic proximity or similarity in the vascular anatomy of the grafting partners does not seem to result always in grafting realization (Wulf et al., 2020). It is shown that grafting is facilitated by the action of genes such as cellulases that help the reconstruction of cell walls; these genes can facilitate tissue adhesion and promote plant grafting (Kurotani et al., 2020; Notaguchi et al., 2020). If plants possess or are bred to possess such genes in abundance, interfamily barriers in grafting could be lifted and grafting technology can be expanded to deliver more chimeric plants that will take advantage of both distantly related partners’ advantages.

Consequently, division and differentiation are key cellular ongoing processes during grafting that require cell communication and hormones involvement. Auxin seems to hold the most important role with the other hormones like cytokinin, ethylene, jasmonic acid (JA), and gibberellin interacting with auxin along the process or participating actively in grafting/wounding responses (Nanda and Melnyk, 2018). Genes coding for auxin efflux proteins were found to be transcriptionally induced in *Arabidopsis* scions, above the graft junctions while auxin response genes are important at the rootstocks below graft junctions, suggesting that auxin may be the signal that is transported from the scion to the rootstock to activate vascular reconnection at the very early stages of grafting (Melnyk et al., 2015, 2018). A study in watermelon grafted on squash has recently shown that the expression levels of most auxin transporter genes changed during grafting enforcing the suggestion that these auxin-related genes play an important role in auxin transportation toward graft junctions, thus promoting wound healing and vascular formation (Yu et al., 2017). Experiments in melon grafted on *Cucurbita* rootstocks have earlier pointed toward this direction as auxin was suggested to act by triggering ethylene production and reactive oxygen species generation, which promotes root degradation in rootstocks that are more susceptible to auxin than others (Aloni et al., 2008).

## Trafficking of Genetic Information Exchange Between the Rootstock and the Scion

**Figure 1:**
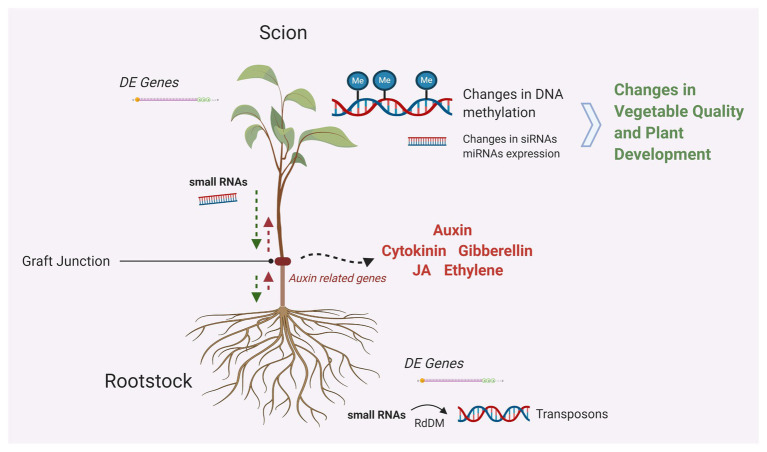
Graphical summary of the molecular mechanisms taking place in grafted plants. Different hormones seem to play a major role in cell division, differentiation, and tissue communication around grafting junctions. Trafficking of genetic information in the form of small RNAs impacts the expression of genes as mobile small RNAs are implicated transposon silencing *via* RNA-directed DNA methylation (RdDM). Changes in DNA methylation are associated with changes in gene expression and impact phenotypic variation (Created with Biorender.com).

We are just starting to understand the molecular processes involved in vegetable grafting. However, the alteration of plants phenotype, development, and performance by grafting has been long documented; one of the terms introduced to explain the diversity observed in grafted plants is graft “hybridization.” A term first introduced by Darwin in the 19th century, graft hybridization refers to the asexual hybridization that occurs during grafting and possibly results to heritable changes (Liu, 2006). Later, Michurin introduced “mentor” grafting – a method of grafting where scions from old tree fruit cultivars are grafted on the lower branches of young trees providing them with characteristics they lacked before. Michurin believed that rootstocks contribute their hereditary materials to hybrids (Liu, 2006). Lots of scientists have used mentor grafting to produce heritable graft-induced variation and graft hybrids. Graft-induced changes on several plant characteristics producing many phenotypic variants have been reported in peppers (Hirata and Yagishita, 1986; Yagishita and Hirata, 1986, 1987; Yagishita et al., 1990; Taller et al., 1998, 1999; Tsaballa et al., 2013). These findings were clearly pointing out toward trafficking of genetic information between the two grafted partners (Figure 1). Indeed, early genetic studies have shown chromatin movements from rootstock to scion and subsequently to the seed progenies (Ohta, 1991). Rootstock-specific random amplification of polymorphic DNA (RAPDs) molecular markers were detected in the graft-induced variants differentiating them molecularly from the scion (Taller et al., 1998).

Recently, Stegemann and Bock (2009) found that in graft sites of tobacco transgenic lines carrying different antibiotics markers and reporters, develop cells resistant to both antibiotics and exhibiting both reporter genes, thus indicating that genetic information is horizontally transferred between the two grafted partners either as DNA pieces or plastids. Later, Stegemann et al. (2012) used callus tissue from the graft sites for plant regeneration originating from reciprocally grafted *Nicotiana* species, being nuclear and chloroplast transformed. They discovered that chloroplast DNA from the *N. tabacum* chloroplast transgenic line has been successfully transferred through the grafting junction to *Nicotiana glauca* or *Nicotiana benthamiana* regardless the *N. tabacum* line being the rootstock or the scion. However, no transfer of nuclear or chloroplast DNA recombination took place. The antibiotic resistance *N. tabacum* plastid genome contributed to the other two nuclear transgenic species was stably inherited to the progenies, a sign of an entirely plastid substitution in the *N. glauca* or *N. benthamiana* lines (Stegemann et al., 2012). Fuentes et al. (2014) have used *N. glauca* scions, genetically modified (GM) to express the *nptII* gene (kan resistance), and *N. tabacum* rootstocks, modified to express the *hpt* gene (hygromycin resistance). The grafting of the two species led to the development of a new plant that was simultaneously resistant to both antibiotics thus harboring both nuclei or both genes through recombination. Although no similar studies have been published in vegetables, the aforementioned work on tobacco might lead the way for similar studies that would possibly elucidate the genetic effects of vegetable grafting on grafted plants’ phenotype.

## Grafting and RNA Transport

Movement of genetic information in the form of small RNAs is a key-issue that has been discussed extensively in literature over the past decade. Messenger RNA (mRNA) molecules have been long known to travel inside plants, *via* the phloem, and grafting has been used in many related studies as a tool to prove exactly this (Lough and Lucas, 2006; Harada, 2010; Spiegelman et al., 2013; Turnbull and Lopez-Cobollo, 2013). For small RNAs, a phosphorus deficiency induced miRNA, the miR399, was the first to be detected in the phloem sap of rapeseed and pumpkin while the use of micro-grafting experiments has shown that it is moving from shoots to roots (Pant et al., 2008). More miRNAs, such as miR156, miR172, and miR395, have been proposed to move from the scion to the rootstock (Martin et al., 2009; Buhtz et al., 2010; Bhogale et al., 2014).

However, it was basic research in *Arabidopsis* that has recently paved the way for a new understanding of the genetic information moving within grafted plants in the form of siRNAs. The predominant heterochromatic 24-nt siRNAs have been documented to direct *de novo* methylation of mainly transposable elements (TEs) and repetitive DNA in a process called RNA-directed DNA methylation (RdDM; Borges and Martienssen, 2015; Xie and Yu, 2015) resulting in transcriptional gene silencing (TGS). Molnar et al. (2010) used wild type (WT) *Αrabidopsis* plants and mutants grafted onto each other to show that several types of sRNAs are moving inside grafted plants but mainly 24-nt siRNAs generated by the action of a Dicer-like protein, DCL3, are mobile and are transported from shoot to root. Although other classes of sRNAs like the 22-nt and 23-nt are also mobile, 24-nt siRNAs were linked to DNA methylation of three specific loci which were TEs in the roots (Molnar et al., 2010). This siRNA movement is carried out by the phloem. Later, it was proved by experiments using *Arabidopsis* transgenic lines that only the mobile 24-nt siRNAs are directing DNA methylation at the recipient meristematic tissues and TGS of a transgene promoter (Melnyk et al., 2011). Finally, it was demonstrated that mobile 24-nt siRNAs – although again not exclusively the only mobile sRNAs class – can travel from shoot to root to direct DNA methylation of thousands of genome sites associated again with TEs (Lewsey et al., 2016).

The mobility of sRNAs inside grafted plants has been proved to be more efficient when sRNAs are produced in the scion and travel toward the rootstock than vice versa, as it is proposed that they move through the phloem and plasmodesmata (Molnar et al., 2010; Melnyk et al., 2011). Still, siRNAs produced in rootstock phloem companion cells were proved to travel toward the WT scion and lessen the viroid infection facilitated by the removal of lateral leaves and buds (Kasai et al., 2013). In tomato transgenic rootstocks with a silenced fatty acid desaturase gene (*LeFAD7*), non-transgenic scions were used for grafting. Grafted scions had lower expression of *LeFAD7* gene and siRNAs were present, indicating that they were transmitted to the scion by the GM rootstock. Scions had their leaves removed before grafting in this work too (Nakamura et al., 2015). Interestingly, earlier studies in pepper where graft-induced changes were recorded in the scions included mentor-grafting (Taller et al., 1998). In mentor-grafting, leaves of the scions are removed to promote the flow of substances from the rootstock to the scion (Goldschmidt, 2014). It is possible that sRNAs generated from the rootstock could also flow toward that direction among these substances and could underline the significant changes observed in scions’ phenotypes.

The apparent movement of sRNAs inside grafted plants could also have vast practical implications. For example, resistance to viruses has been shown to be transmitted from the rootstock to scion in tomato. Spanò et al. (2015) managed to produce resistant to TSWV scions that accumulate less viral RNA when grafted on a resistant tomato variety that has stronger RNA interference (RNAi) response to the viral infection. By qRT-PCR analysis, it was shown that the expression of essential genes of the RNAi mechanisms, such as Argonaute (AGO) and RNA-Dependent RNA polymerase (RDR) genes, was upregulated in the roots of resistant grafted plants. Interestingly, the authors found that RNA silencing was also stronger in the self-grafted plants showing that even grafting itself could provoke the activation of the mechanism (Spanò et al., 2015). Furthermore, if one of the two grafting partners is GM, then we can have a new plant breeding technique (NBT) in our disposal. As a result, the other part’s fruits or flowers, often the scion, will not be considered GM since the produce comes from the plant part that is not GM (usually the scion) clearly benefiting from the genetic modification advantages of the other grafting partner. This NBT technique also called “transgrafting” (Haroldsen et al., 2012), it is based on the mobility of various genetic material that can travel inside grafted plants and has already been documented (Bai et al., 2011). Works that prove the effect of a transgenic rootstock in a non-transgenic scion could pave the way for an agricultural product generated through transgrafting and at the same time be free of any GM related technology implications. Given Europe’s caution toward GM technology and products and the recent ruling of European Court of Justice on gene editing, including CRISPR-modified crops being subjected to GMO regulations, plant science and agriculture should carefully explore the possible use of grafting using resistant rootstocks or even transgrafting to produce agricultural products of higher yield and quality.

## Grafting and Epigenetics Mechanisms

Three main epigenetic mechanisms have been described in plants; DNA methylation/demethylation, histone modifications, and non-coding RNA mediated action, all either activating or silencing plant genes (Kapazoglou et al., 2018). Plant DNA methylation, the process that involves the addition of a methyl group (CH_3_) to DNA cytosines resulting in a 5-methylcytosine, occurs in CG, CHG, and CHH backgrounds (He et al., 2011). Non-coding RNA and more specifically small non-coding RNAs or sRNAs of 21–24-nt, the result of the specific action of Dicer-like (DCL) proteins, are targeting homologous gene transcripts to either cleave them or repress/inhibit their translation. There are four classes of small RNAs in plants; micro-RNAs (miRNAs) that are post-transcriptional regulators while the other three classes (often collectively referred as siRNAs) are additionally involved in transcriptional gene silencing. siRNAs which have a length of 24 nt are implicated in transposon silencing by RNA-directed DNA methylation mechanisms (Chen, 2009; Molnar et al., 2011).

In grafting, epigenetic effects have been associated with DNA methylation and non-coding RNAs. Changes in DNA methylation have been observed in grafted plants on several occasions. Although one could argue that this is not surprising since changes in DNA methylation have been well evidenced to be linked with stresses such as cutting and wounding (Cao et al., 2016), it seems that plant grafting and DNA methylation relationship goes beyond wound stress. A study showing changes in DNA methylation in grafted Solanaceae plants was published by Wu et al. (2013), who used tomato and eggplant grafted onto each other while pepper was used only as a rootstock for tomato. Methylation-specific amplified markers (MSAP) analysis revealed no changes in global methylation of the grafted plants. Only changes in local methylation were detected, in a locus-specific way, in the scions and pepper rootstocks. A large portion of these DNA methylation changes in scions was inherited in the self-pollinated grafted progenies. Further bisuflite sequencing (BS) of specific loci has confirmed that even though self-grafting can produce some DNA methylation changes, interspecies grafting in Solanaceae is accompanied by significant heritable cytosine methylation changes. Specific genes all linked to DNA methylation, such as *Methyl Transferase (MET) 1*, had significantly altered expression profiles in tomato to eggplant grafted plants, although these profiles were reversed in the progenies, in comparison to their seed non grafted controls (Wu et al., 2013). This study was one of the first that directly implicates epigenetics and DNA methylation in grafting, especially in scions. Our work on Cucurbitaceae grafting also revealed that DNA methylation might be implicated in grafting effects in other plant families, where interspecies grafting is common practice. Specifically, by grafting cucumber, melon and watermelon on pumpkin and using MSAP markers, we have recorded a significant rise of global DNA methylation in cucumber and melon scions but interestingly not in watermelon (Avramidou et al., 2015). This suggests that epigenetic alterations occurring in grafting scions could be specific of the interaction between the rootstock and the scion.

Important work on epigenetics and grafting was also conducted in Brassicaceae plants. Ιnter-species *in vitro* grafting that involved the union of two vertical cut plantlets of tuber mustard (*Brassica juncea*) and red cabbage (*Brassica oleracea*), treated with cytokinin and auxin before grafting, resulted in a periclinal chimeric shoot apical meristem (SAM) comprising of layers from both species; layers I and II from tuber mustard and layer III from red cabbage, resulting in plants having characteristics from both parents (Chen et al., 2006). MSAP methylation status analysis of the seed progenies after selfing the grafted chimera, revealed significant changes in DNA methylation compared to the non-chimeric non-grafted plants. Interestingly, some of the DNA methylation changes were retained for five generations after grafting, while others were reversed. Phenotypic characteristics such as SAM termination and early flowering were also reversed while other changes, e.g., in leaf shape were stably inherited in these five generations. Most of the differentially methylated fragments were found to belong to transposons (Cao et al., 2016). Transposons are often silenced by DNA methylation in plants (Lippman and Martienssen, 2004). Interestingly, it has been revealed that siRNAs were differentially expressed in three progeny selfing generations after grafting. Some 24-nt siRNAs involved were mapped to the differentially methylated fragments while their expression was decreased in leaves of the chimeric grafted progenies in comparison to the leaves of non-chimeric plants (Cao et al., 2016). It was previously suggested that specific siRNAs from red cabbage are present in the leaves of another chimera’s tuber mustard/red cabbage progenies reverted to their tuber mustard genotype, showing that transfer of siRNAs from one species to the other is possible between grafted partners. In this study, leaf shape was again stable while SAM termination was not. Furthermore, some miRNAs had different expression patterns in the reverted progenies in comparison to the non-chimeric controls and these patterns were accompanied by expression variations of their target genes (Li et al., 2013).

In *Cucurbita pepo*, we have used intra-species/inter-cultivar grafting to study the effect of grafting on scion fruit quality and we performed methylation and miRNA studies to monitor the fruit phenotypic changes happening after grafting. We concluded that *Cucurbita* grafting alters global DNA methylation patterns and expression of specific miRNAs, as evidenced by MSAP and qRT-PCR analyses (Xanthopoulou et al., 2019). Li et al. (2014) carried out reciprocal graftings of cucumber and pumpkin as well as homo-graftings. They sequenced RNA from leaves and root tips of the grafted plants and compared the expression of miRNAs in the hetero-grafts and the homo-grafts. They found that the expression of most of the miRNAs has changed in the hetero-grafted in comparison to the homo-grafted (Li et al., 2014). However, the expression of miRNAs in grafted plants cannot be considered separately from stresses. Different miRNA expression patterns in homo- and hetero-grafted cucumbers grafted onto pumpkin rootstocks were detected under salt stress showing that miRNA regulation might be a result of salt stress adaptation (Li et al., 2016). Whether miRNAs are the cause or the result of stress adaptations and whether there is a direct involvement of different rootstock and scion combinations into these effects are yet to be determined.

Mobile epigenetic signals might have huge implications in species routinely grafted like pepper and cucumber. In other species of the Solanaceae family, sRNA movement was evidenced using transgenic *N. benthamiana* scions producing siRNAs grafted on potato rootstocks that either express green fluorescent protein (GFP) or not. SiRNAs were found to be able to transcriptionally silence GFP in potato lateral roots accompanied by hypermethylation of the target region (Kasai et al., 2016). Roots tissue culture produced regenerated shoots and micro-tubers that manifested TGS of GFP. High methylation levels in the endogenous *granule-bound starch synthase I* (*GBSSI*) gene target region were detected in two micro-tubers on the adventitious shoots formed from *N. benthamiana* transgenic scions grafted on WT potato rootstocks. The shoots produced by these micro-tubers maintained the high methylation status and so did the second progeny tubers without any siRNA presence suggesting that the characteristic was stable inherited (Kasai et al., 2016).

The connection between the already observed and documented phenotypic grafting effects and sRNAs-associated DNA methylation and how this could result in gene expression changes that in turn can lead to the phenotypic grafting effects is extremely interesting. It is also intriguing to think about the other classes of sRNAs that are considered mobile like the 21-nt miRNAs, directly linked to developmental changes, growth and stress responses in plants. In addition, canonical miRNAs of 20–22-nt have been shown to cause DNA methylation (Bao et al., 2004) as well as longer non-canonical miRNAs (Wu et al., 2010). Mobile sRNAs that move through the phloem and grafting junctions, following the source to sink gradient, could lie behind heritable epigenetic changes recorded in the grafting partners, opening a new potential for the use of grafted plants in plant breeding. These epigenetic changes could possibly involve changes in DNA methylation as it was discussed above. However, the origin, movement, and involvement of these sRNAs in epigenetic changes need further exploration. Finally, to our best knowledge, until today, there has not been any study linking vegetable grafting and histone modifications, the third known epigenetic plant mechanism.

## Grafting and Changes in Gene Expression

Since grafting changes a plants phenotype, gene expression and molecular mechanisms that are implicated in controlling gene expression are also thought to be altered. Many questions regarding grafting and gene expression changes have been raised. Do Gene expression alterations occur due to the grafting process *per se*, a process that causes significant wounding stress and entails rootstock/scion recognition and interaction? It is known that grafting triggers different mechanisms apart from wounding. When specific RNA-sequencing (RNAseq) was performed on grafted parts 0.5 mm above and below the graft junction and compared with cut, separated but not grafted scions and rootstocks, (Melnyk et al., 2018) found specific genes, such as immune- and chitin-responsive but also vascular growth genes that are uniquely expressed during grafting. Cookson et al. (2014) carried a similar work in grapes by grafting 1 m long stems of a grape cultivar scion onto two different cultivars-rootstocks. Expression differences were identified between the hetero-grafted and the homo-grafted in genes related to oxidative stress, receptor kinases, JA signaling, cell walls, lignin biosynthesis, and pathogenesis related proteins. Other questions revolve around the influence of the rootstock on the scion and vice-versa that makes the difference in gene expression. What is the effect of inter-species vs. intra-species grafting on gene expression; do taxonomically different species of diverse genera have a larger or smaller transcriptomic effect when grafted in comparison to same genus grafting? Answers to these questions are not that simple, revealing the complexity of the issue.

Again, work in *Arabidopsis* proves indispensable for drawing valuable conclusions and paving the way for other species regarding grafting. *Arabidopsis* plants were homo-grafted into each other and microarrays analysis was used for detangling the expression of genes in the same organs of rootstock and scion, flower buds, and leaves that were left to grow after grafting on each of the grafting partners. It was found that grafting *per se* can cause different responses in the expression of scion and rootstock genes ranging from those controlling biotic and abiotic stress responses to transcription factors, flower development, and hormones pathways (Kumari et al., 2015).

More studies evolving around reveal global, comprehensive, and often detailed transcriptome changes in hetero-grafted plants. Garcia-Lozano et al. (2020) investigated the transcriptomic changes in watermelon scions grafted on bottle gourd (*Lagenaria* spp.) and the reciprocal graftings along with their homo-grafted controls. Numerous genes related to different aspects of ripening and quality or even stress response, were found to be differentially expressed in tissues of both grafting combinations as compared to the homo-grafts. Furthermore, it was found that more than 400 mobile mRNAs are present in both heterografts the majority of which move from rootstock to scion (Garcia-Lozano et al., 2020). In a previous study, watermelon scions grafted on bottle gourd had much less differentially expressed genes (DEGs) than watermelon grafted on squash in comparison to the homo-grafted watermelon to watermelon control. In addition, the identified DEGs were shown to belong to several different gene ontology (GO) categories ranging from metabolism to environmental responses, from those 49 genes encoding for pentatricopeptide repeat and WD40 proteins were induced only in the watermelon to squash grafting combination (Liu et al., 2016a). This indicates the effect of rootstock’s genotype on the scion’s transcriptomic profile. However, the fact that thousands of genes are induced in both inter-species grafting combinations in comparison to the homo-grafted controls also shows the effect of hetero-grafting. A similar study was performed in cucumber where analysis of the fruit mesocarp’s transcriptome was performed. It was found that when cucumber is grafted onto *Cucurbita ficifolia* and *Cucurbita moschata* hybrids, genes that affect fruit quality such as those participating in the production of sugar and aromatic compounds are differentially expressed between grafting combinations (Zhao et al., 2018). The choice of rootstock can affect not only scion’s growth, yield, and tolerance to stresses but also efficiently affect genes that control quality. Yet, the effect of grafting *per se* is severely depicted by the differentially expressed genes associated with fruit quality and reported in grafted plants (Aslam et al., 2020).

The effect on quality is the reason for many grafting studies exploring possible rootstock or grafting effects on scions. We have extensively studied the effect of grafting on fruit quality in pepper. Having observed and recorded inheritable phenotypic changes in scions’ fruits shape and having identified *CaOvate* gene as a possible fruit shape regulator in pepper (Tsaballa et al., 2011) we attempted to associate the inherited changes in fruit shape in pepper grafted progenies, retaining the change in fruit shape, to this particular gene. We have recorded an increase in the expression of *CaOvate* gene in the ovaries of plant’s progenies retaining the fruit shape change recorded in grafting, in comparison to the two grafting partners. This might imply that grafting between cultivars of different fruit shape might have caused expression changes in *CaOvate* which are maintained in the progenies (Tsaballa et al., 2013). A very recent report regarding grafting of tomato on potato is showing an important aspect previously observed only in trees. Potato rootstock induces only minor phenotypic changes onto tomato scions having small effect on DEGs in comparison to homo-grafted controls. Nevertheless, at the same time tomato scions have a major effect on potato rootstocks causing the differential expression of thousands of genes some of them involved in hormone signaling and hormone pathways (Zhang et al., 2019). The effect of grafting onto the underground root protein profile was also evidenced in watermelon grafted on bottlegourd. Grafting was found to enhance the diversity of the proteins released by roots in comparison to non-grafted plants and most of these proteins are implicated in biotic and abiotic stress resistances (Song et al., 2016).

The transcriptomic nature of rootstock-induced resistance has also been the subject of many studies in grafted plants exposed to stresses. This was shown in tomato where a popular cultivar was grafted on two different rootstocks, one cultivar susceptible to cold and a wild species (*Solanum habrochaites*) tolerant to cold. These graftings resulted in only a few hundreds of genes differentially expressed in the leaves of the scion when grafted on the sensitive rootstock in comparison to the tolerant one. Still thousands of genes are differentially expressed in the two rootstocks in grafted plants exposed to sub-optimal temperatures, mainly genes involved in defense mechanisms (Ntatsi et al., 2017). This implies that often the acquired resistance to stresses in grafted plants is attributed to graft-induced changes in the root system. Changes in the expression of genes in tomato scions can be recorded early in the grafting process. Wang et al. (2019) showed that tomato heterografts and homografts have similar healing profiles and the examination of gene expression in tomato scions, 16 days after grafting, revealed many DEGs including those related to signaling and oxidative stress that were upregulated in the heterografted scions (Wang et al., 2019).

However, grafting *per se* could be responsible for the acquired resistance to stresses. A study including transcriptomic analysis in tomato has proven that grafted plants can recover from potato virus Y (PVY) infection independently of whether a susceptible or a tolerant variety is used as rootstock or scion. It appears that in the case of the tolerant variety, its favorable tolerance to viral infection is augmented by grafting changing its transcriptome, while it can be also effectively delivered to a susceptible scion (Spanò et al., 2020). It appears that tolerant varieties with their special coping mechanisms against viral infections can act cumulative and/or synergistically in grafting (Spanò et al., 2017). Moreover, grafting itself can further influence scion characteristics. When sweet potato (*Ipomoea batatas*) was grafted on Japanese morning glory (*Ipomoea nil*) flowering was induced in the otherwise non-flowering sweet potato. Transcriptomic analyses suggested that all genes in the grafted stems that participate in the anthocyanin biosynthesis pathway as well as genes involved in the induction of high ethylene levels in grafted flowers were induced in comparison to the non-grafted controls. Thus suggesting that grafting and its stress-induced conditions can significantly alter development characteristics in vegetables (Wei et al., 2019).

## Conclusion

Grafting interactions are complex. Emerging research studies show that grafting changes gene expression that impacts scion’s phenotype. It also suggested that often the interaction of genotypes has a significant impact on grafted plants performance. It seems that this specific interaction forms certain transcriptomic patterns on grafted plants that often lead to a total reprogramming of gene expression. However, the effect of grafting as a process on gene expression cannot be neglected. What is essential for future studies on the exploration of molecular mechanisms that control grafting impact and interaction is that in every grafting experiment these aspects should be examined separately using the appropriate controls and careful examination of RNAseq data.

Through the advent of high throughput sequencing many reference genomes are now known and available for a variety of plant species. Using modern molecular techniques and the vast information available on bioinformatic databases, it is now possible to obtain a deeper understanding on the genomic interactions that take place during grafting. It is also possible more now than ever, to elucidate further the molecular aspects that facilitate grafting establishment, communication, and movement of genetic information inside grafted plants. Resolving compatibility issues could help the expansion in the use of grafting in more plant species contributing to sustainability and preservation of biodiversity. The plastic epigenome of grafted plants needs to be further explored to comprehend the impact on the phenotype and performance of grafted plants and steer the technology toward utilizing it for its advantage. Epigenetic diversity could act as a new source of phenotypic variability, thus helping toward adapting to the environment that is being transformed (Gallusci et al., 2017). Mapping of epigenetic marks on plant genomes and the identification of epigenetic targets could provide breeders with new means to increase and use epigenetic variability in their efforts for breeding new crop varieties (Varotto et al., 2020). In the future, the deepening of our knowledge on plant grafting could lead us to take advantage of all the possibilities plant grafting is offering or even manipulate it in favor of agricultural production.

## Author Contributions

All authors listed have made a substantial, direct and intellectual contribution to the work, and approved it for publication.

### Conflict of Interest

The authors declare that the research was conducted in the absence of any commercial or financial relationships that could be construed as a potential conflict of interest.

## Footnotes

^1^www.vegetablegrafting.org
